# Interpreters in UK Asylum Appeals: Evidence and Impact

**DOI:** 10.1007/s11196-025-10386-6

**Published:** 2025-12-08

**Authors:** Alice Richardson

**Affiliations:** Department of Language and Linguistic Science, University of York, York, UK

**Keywords:** Asylum, Legal interpreting, Language as evidence, Asylum and refugee interpreting, Access to justice, Credibility assessment

## Abstract

This paper considers the role of interpreters in the asylum system in the UK. The study analyses published reports of asylum appeals in the UK from 2000 to 2024, and considers how interpreters are referred to by judges, and how they impacted the outcome of the appeal. Adopting a mixed-methods approach, the study initially takes a broad quantitative view of the issue, followed by a Qualitative Content Analysis of 30 recent appeals. The results reveal that interpreters are materially impacting asylum outcomes, and at times, interpreting issues form the central motivation for lodging an appeal. The type and impact of interpreter interventions range from procedural to critical, with critical cases calling into question the applicant’s credibility—a vital component of an asylum claim—which has lasting effects throughout the entire case. The findings raise questions about the current provision of interpreting services in UK asylum appeals, and how interpreter performance and comprehension can have significant legal ramifications on case outcomes. The findings contribute novel evidence to the current literature on the role of language in access to justice.

## Introduction

There were over eight million people seeking asylum globally in 2024 [1]. This paper examines the role that interpreters play in the protracted legal battle of an asylum application. The asylum system entails the meeting of different cultures, languages, and institutional settings in a fact-finding mission which relies heavily on oral communication [2–6]. The system would not function without interpreters. In the UK, the sociopolitical context is such that the asylum-seeker must pursue their application within a culture of disbelief, thanks to an increasingly hostile environment generated by governmental policy and mainstream media [7–9]. Not only must asylum-seekers contend with barriers to social integration and prejudiced treatment, but they must also navigate a legal system which is explicitly designed to ‘catch them out’ and to uncover apparent inconsistencies in their story [10–12].

**Fig. 1:**
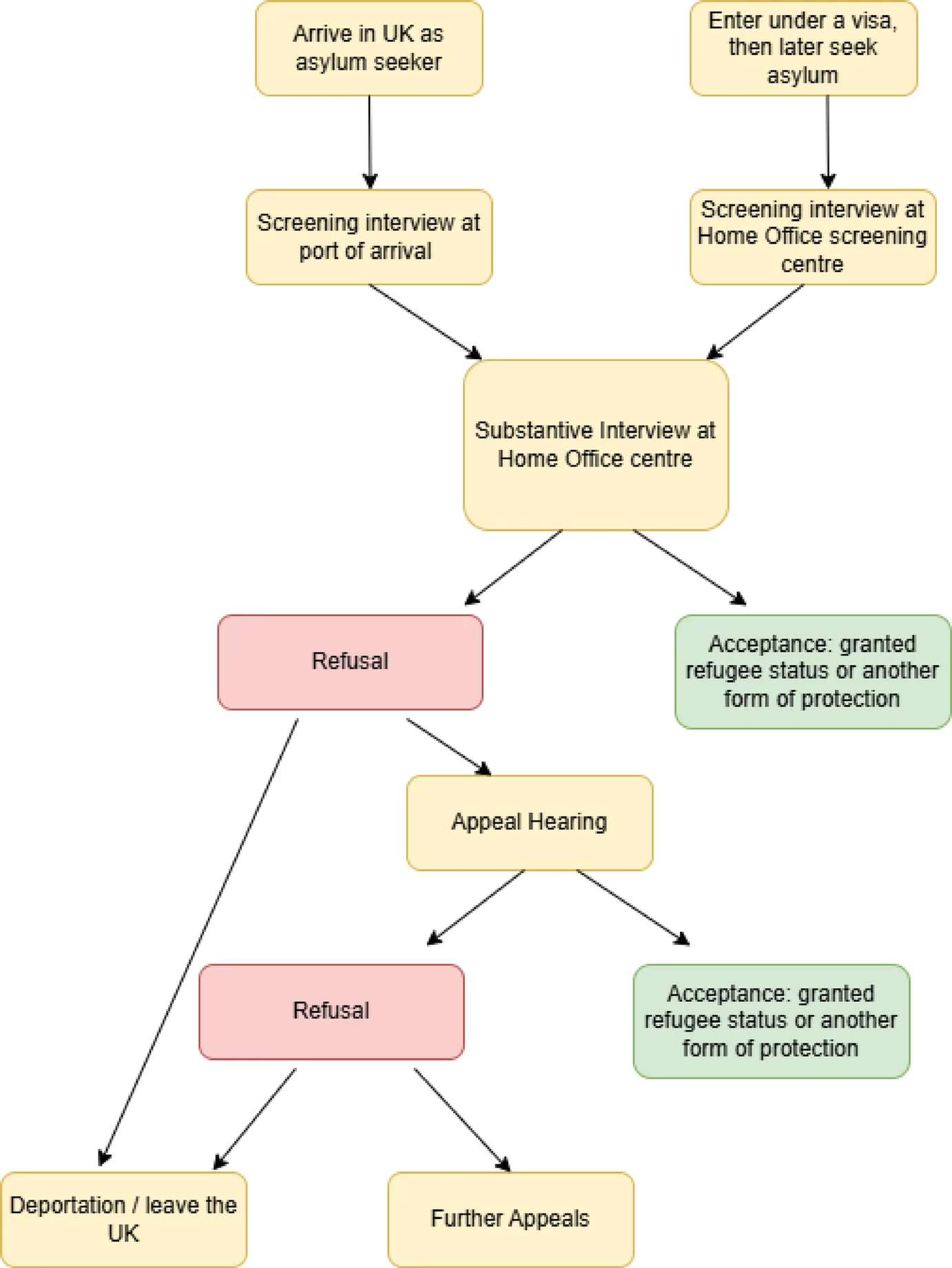
Flowchart illustrating the timeline of seeking asylum in the UK

Seeking asylum in the UK involves multiple interactions with the authorities. There are three key stages: the screening interview, the substantive interview, and an appeal hearing, as illustrated in Fig. 1. The screening interview is brief, and aims only to gather basic facts about the applicant. The substantive interview is where the applicant outlines their circumstances, history, and the reasons why they are seeking asylum. According to Home Office figures from 2024, 52% of initial decisions after a substantive interview are refusals [13]. The majority of those who receive a refusal notice will then lodge an appeal [14]. Appellants will attend an appeal hearing at an immigration tribunal, and, depending on the outcome, may lodge further appeals.

Throughout each interaction, the applicant is tasked with proving to the decision maker that they have a “well-founded fear of persecution,” as defined by the 1951 Convention Relating to the Status of Refugees [15]. The most vital asset to an asylum-seeker, though, is credibility. Demonstrating to decision makers that they are a credible witness is the biggest and most important task of the asylum applicant [16–19]. There is an ‘inconsistency ideology’ [17] pervasive in refugee determination procedures: the asylum-seeker must appear consistent throughout to appear credible and to succeed. It is therefore crucial that the asylum narrative appears consistent to decision makers across each interaction [20].

At each stage of the process, an interpreter is provided for the applicant at public expense, as stipulated by the immigration rules [21, 22]. The role of interpreters in allowing the asylum-seeker to tell their story is so critical, and so inextricably linked to Human Rights, that interpreting itself becomes a right [3]. In the UK, the provision of interpreting services is overseen by various agencies (see Sect. 2.1), meaning that asylum-seekers are likely to have a different interpreter for each interaction. This creates the scenario whereby two interpreters might offer equally valid but different translations, leaving the applicant vulnerable to potential *perceived* inconsistencies [23]. This is by no means the fault of the interpreter; translation is not a mathematical equation, and the most expert, qualified, and skilled interpreters might offer subtle variations in the rendering of a word or tense, for instance.

More concerning, though, are recent reports which have revealed a reduced quality of interpreting in the asylum process, particularly since a decision to outsource the provision in 2011 [24] (see Sect. 2.1). This reduction in quality puts asylum applicants at an unjust disadvantage; an apparent inconsistency and its damage to the applicant’s credibility could potentially have devastating consequences for their asylum application, and thus their life [25]. This paper therefore will consider what empirical data reveals about the extent to which interpreters affect the outcomes of asylum applications.

**Fig. 2:**
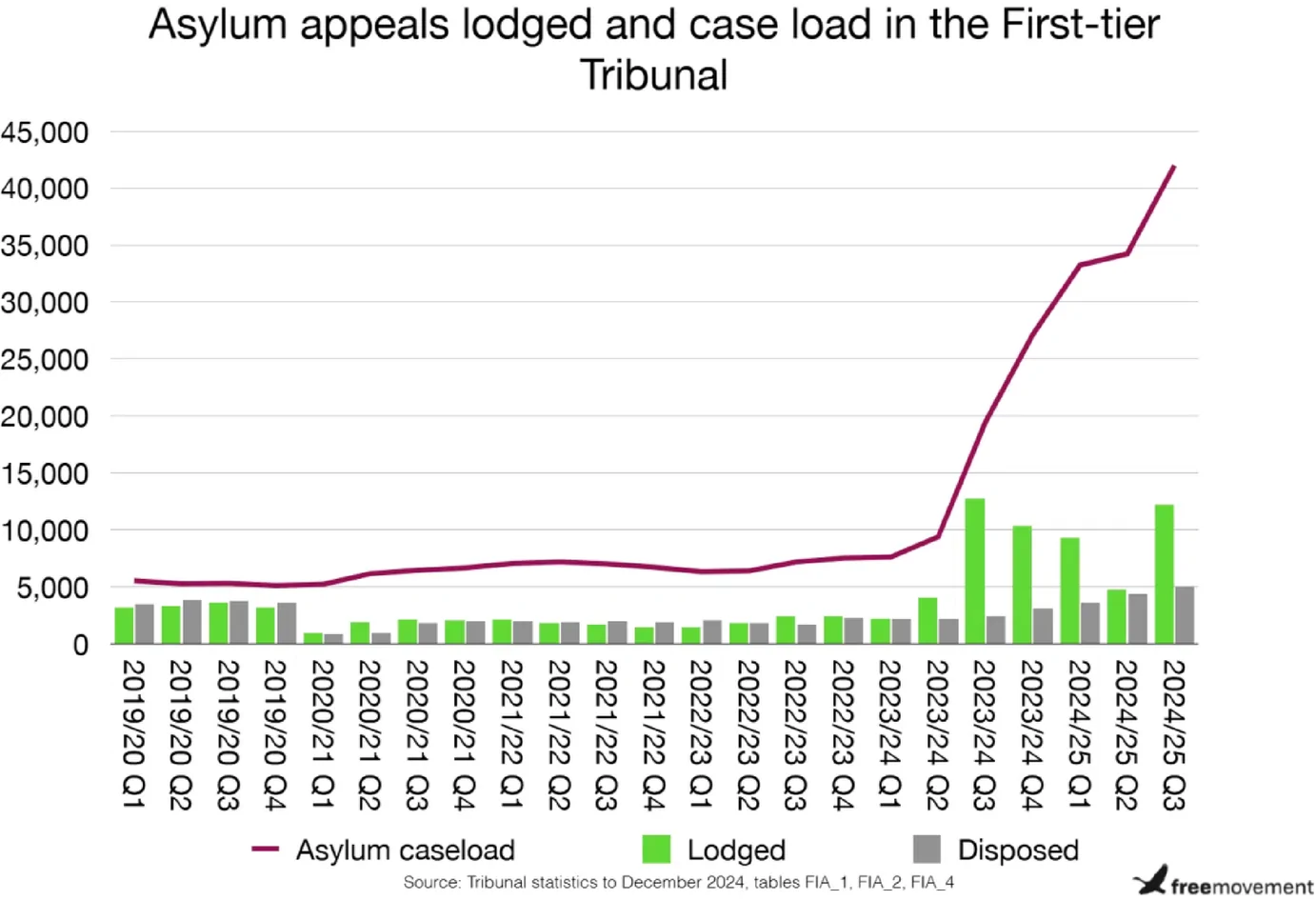
Line graph showing number of asylum appeals lodged in 2024 [27]

This investigation focuses solely on the appeal stage of an asylum application, for several reasons. First, credibility is yet more important at the appeals stage, where it becomes the very core of the process in the absence of a concrete legal framework [7, 16] (see Sect. 2.3). In addition, recent political events including the establishment of the Nationality and Borders Act 2022, the Illegal Migration Act 2023, and the government’s pledge to clear the backlog of asylum claims, have all led to a sharp rise in asylum decisions being made. This in turn has caused a dramatic increase in the number of appeals being lodged, with appeal rates reaching an all-time high [26]. Somewhat paradoxically, the push to fast-track decisions and reduce the number of applications has triggered a higher-than-usual volume of appeals based on human-rights violations [27]. In addition, cuts to legal aid and a reduction in initial positive decisions from the Home Office have also increased appeal rates (of which almost half of all appeals were successful at their first stage, suggesting inadequate decision-making following substantive interviews) [28, 29]. The upward trend in appeals is illustrated in Fig. 2.

Almost 27,000 appeals were lodged in the first nine months of 2024, representing a 71% increase compared to 2023 [30]. This sharp increase highlights the importance of investigating interpreting from an appellate perspective. To explore what role interpreters play in the asylum appeal process, this paper will answer the following research questions:

1. In what ways do judges refer to interpreters in Decision Notices?
2. Are interpreters having an impact on appeal outcomes? If so, in what ways?

To answer these research questions, a quantitative summary was combined with a finer-grained qualitative analysis. The subject of analysis was Decision Notices (DNs). These are reports written by judges after an upper-tier tribunal (first-tier DNs are not available to the public). A DN recounts the history of the case, as well as a summary of the appeal hearing and the final decision. DNs are an extremely useful source of information; they protect the anonymity of appellants, and are rich with data that is normally extremely difficult to access due to its sensitive nature. They are also written by judges, a participant group whose perspective is notoriously difficult to access for research purposes. Whilst there is substantial socio-legal research considering the legal framework of the asylum appeal process, and an ever-growing body of work dedicated to interpreters in the asylum process more generally (see for example: [8, 16, 28, 31–33] (see Sect. 2), there is scant attention regarding the role of interpreters from an appellate perspective. Furthermore, to my knowledge, at the time of writing, there are no studies at all which have investigated decision notices.

## Background and Literature

### Institutional Context

The provision of interpreting services is managed by different governmental bodies for each stage of an asylum application. For screening interviews, the UK Border Force sources interpreters either through the Interpreter & Language Services Unit (ILSU) managed by the Home Office, or from an agency, which is *thebigword* at the time of writing (in some instances they will alternatively rely on emergency provisions). For substantive interviews, ILSU sources interpreters either through their own in-house list, or through *thebigword*. For appeal hearings, interpreters are provided by the Ministry of Justice (MoJ) who primarily use *thebigword,* with whom they have a contractual agreement, following the outsourcing of this service in 2010 (See [34] for a summary).

Prior to the outsourcing, the MoJ relied on the National Register of Public Service Interpreters (NRPSI) to source interpreters for asylum appeals [23]. To be a member of the NRPSI, interpreters must hold a diploma in public service interpreting, and pay a membership fee. Following the move to *thebigword,* however, interpreters with fewer skills and qualifications were allowed to work for the MoJ, particularly if they are speakers of ‘rare’ languages [35]. Furthermore, interpreters’ working conditions and rates of pay reduced drastically. Many professional interpreters therefore boycotted the new framework and are still doing so today [20, 23, 33, 36–40]. The decision to outsource was controversial, and its ramifications are still being debated [41], but a full exploration of the issue is beyond the scope of this paper. It is sufficient to say that following parliamentary debate, the decision to keep the service outsourced has been upheld, and that the contract is up for tender in 2024. This ongoing, contentious matter is even more reason to dedicate research attention to the appeals stage of an asylum application.

### Relevant Literature

The role of the interpreter in asylum proceedings as an area of research, whilst still relatively small, has been gaining traction in recent years, both in the UK and elsewhere. Several scholars have highlighted the key role that interpreters play in asylum determination systems. For instance, Lee’s [42] courtroom observation revealed the damage caused to South Korean asylum appeal hearings by untrained ad hoc interpreters who frequently altered testimony. Khan [43] offers a South African perspective, highlighting how the government’s failings in providing adequate interpreting services are preventing people from accessing their rights. Berbel’s [44] recent exploration of the situation in Germany reveals a need for increased training and auditing of interpreters to ensure quality. Ample work in Belgium [6, 45], Austria [2, 46], France [23, 47] and Australia [31, 48, 49] also consistently highlights how interpretation mediates asylum narratives, suggesting systemic challenges in ensuring linguistic access to justice across jurisdictions.

Key themes in the literature centre on the role of the interpreter, both in the interaction and also the overall asylum process. Eades [50] outlines several language ideologies which pervade legal procedures. According to Smith-Khan [51], the asylum system relies heavily on four such ideologies: the ideology of inconsistency (the core goal is to ‘hunt’ for consistency between enactments of a narrative), the ideology of narrator authorship (the view that the asylum-seeker has created the testimony alone and without intervention), the ideology of decontextualized fragments (the practice of extracting words and phrases from their overall context to consider their validity), and the ideology of entexualisation (the notion that oral testimony can be accurately converted into written testimony, and then taking that written testimony as the official record for comparison).

#### Interpreters’ Roles and Responsibilities in Asylum Hearings

Research highlights the extremely contentious space that interpreters must occupy when at work. Interpreters are tasked with being the go-between for different social and legal actors, all of whom place conflicting demands on them, and who often fail to understand and respect their position [2, 4, 23, 34, 52–54]. The prescribed role of an interpreter is often presented in codes of conduct and by legal professionals as one of an impartial, accurate, and neutral conduit; in other words, a ‘word-machine’ [23, 54–56]. In reality, though, this is an impossible feat: interpreters are active participants who co-create the produced narrative, and must regularly shift between different expected roles [23, 25, 45, 57–59]. Interpreters are prevented from being impartial by demands from interactants [25, 53], attempts to save face [45], or the very nature of their responsibilities. Many cultural concepts do not have exact equivalent translations, and cultural differences might unfairly make the asylum account appear less credible to officials [12], so interpreters are required to step in to mediate discrepancies [23, 54, 60]. It is helpful to view asylum interactions as ‘conversation triads’ [61] rather than translated dyads; interpreters are active participants in an interaction.

Interpreters might also actively fight against the prescribed ‘language machine’ role by advocating for the asylum-seeker, trying to lessen their disadvantage by helping them navigate the confusing legal situation and its unique set of demands [5, 12]. On the other hand, interpreters may forego impartiality to fulfil their own interactional goals, such as that of assisting the interviewer in the decision-making process. For instance, Díez [62] observed an interpreter at a Spanish asylum hearing actively contributing to the asylum-seeker’s credibility assessment by inserting their own questions and omitting information, assuming the role of a second interviewer.

Interpreters’ visibility in the interaction is modified by their choice of speech mode. Interpreters might choose to switch between direct and reported speech, i.e. first or third person speech [45]: e.g.: “he said he put it in the bag” vs “I put it in the bag”.Wadensjö terms this ‘relaying by replaying’ or ‘relaying by displaying’ [58]. Replaying is often seen as the preferred, and more professional option, and is often stipulated in codes of conduct, but it is not always chosen by interpreters [58, 63], which can cause problems for the ‘ideology consistency’ [49, 50]; if different interpreters choose replaying and displaying, this will alter the authorship of events in the narrative.

There is a large body of work examining interpreters in criminal courtrooms, which has relevance to the asylum appeal setting. For instance, Berk-Seligson’s [64] ground-breaking research on bilingual courtrooms revealed that interpreters have a significant influence on how jurors perceive speakers. The addition or omission of features such as politeness markers or hedges had an impact on how convincing and trustworthy speakers were perceived to be.

Furthermore, the mere presence of an interpreter can also influence the credibility assessment. Blommaert [65] highlights a ‘monoglot ideology’ whereby immigration authorities assume a clear mapping between nationality and language ability, ignoring the multilingual reality of millions of people’s lives who move across borders. The request for a specific language of interpretation could thus be used against an asylum-seeker as material evidence to discredit their claims of origin.

#### Narrative Demands

As a result of the ‘decontextualised fragments ideology’ [17, 49, 50] the asylum narrative must be moulded into an institutionally appropriate format; the speaker is not afforded storytelling rights over their own account, but asked instead to segment it according to the stipulations of the interview process [4, 66]. Applicants’ legal counsel must possess a set of translation skills themselves, to transform the individual asylum narrative into a suitable legal claim [32, 66–68]. It is important to acknowledge that this ‘invisible’ layer of translation occurs simultaneously with the visible—though just as complex and nuanced—process of oral interpreting between two languages. Interpreting does not occur in a vacuum; it must function within the already layered and dynamic setting of an immigration tribunal. As such, interpreters might modify speech to help shape the narrative according to this ideology, by segmenting it, altering the formality, or correcting perceived grammatical errors [23].

#### Interpreters and the Record

The written record is revered throughout the process, and viewed as a factual recording of each interaction. This is the ‘entextualisation ideology’ [17, 41, 49, 50]. The transcripts from the screening and substantive interviews are dissected in the search for consistency, implying that the conversion from oral testimony to written word is seamless and perfect, which is not the case [42, 43, 66]. Interpreters make significant contributions to the written record, at times modifying the flow of conversation so that it can be documented more easily, or altering by adding or omitting, so much so that they shape and create the narrative produced for the record [57, 66].

Furthermore, interpreters are rarely acknowledged in the written record, with transcripts recorded only in the target language, leaving no trace of any translation occurring [50, 69] (in Sect. 3.2 we will see how often they appear in DNs). Interpreters should be more visible in official records and interview transcripts, so that any interpreting issues which do arise might be handled more justly [50]. This need is closely related to the ‘narrator authorship ideology’ [17, 49, 50] which ignores the co-created nature of the asylum narrative and instead assumes the applicant created it themselves, in a vacuum without external input or influence. Again, this risks unfair damage to the applicant’s credibility, if the real influence of interpreters or other interactants such as interviewers, is not acknowledged.

#### Interpreter Wellbeing

Better support is also essential for interpreters in asylum proceedings, who bear significant emotional burdens while performing their linguistic duties. Research shows that interpreters develop various coping strategies in high-stakes institutional contexts, with effectiveness largely dependent on training and institutional recognition [70]. This emotional dimension remains overlooked despite its potential impact on interpretation quality and legal outcomes [25].

#### Interpreter Qualifications

In England and Wales, to work as an interpreter in asylum appeals, the MoJ stipulates that interpreters must possess certain qualifications according to the type of assignment and the language spoken. In 2025, the MoJ, along with Her Majesty’s Courts and Tribunals (HMCTS) commissioned a report titled the ‘Independent Technical Review of Qualifications and Experience Requirements for the Provision of Spoken Language Interpreting’ [71] which expresses concerns about the complex tiered qualification system.

The qualification framework differentiates between 46 core (or ‘standard’) languages, and 152 ‘rare’ languages [71]. For core languages, interpreters working on ‘complex’ assignments (including asylum appeals) must hold a level 6 Diploma in Public Service Interpreting (DPSI), or equivalent level 6 qualification, plus have 100 h of interpreting experience. However, interpreters who speak ‘rare’ languages are only required to have 50 h of experience, and no formal qualifications whatsoever [71] substantial risk to quality and due process” [71], particularly given that several of the languages classified as rare are from asylum-seeking populations, for example, Tigrinya (from Eritrea), Amharic and Oromo (from Ethiopia), and Kurundi (from Burundi).

### The Appeal Process: Setting and Logistics

**Fig. 3:**
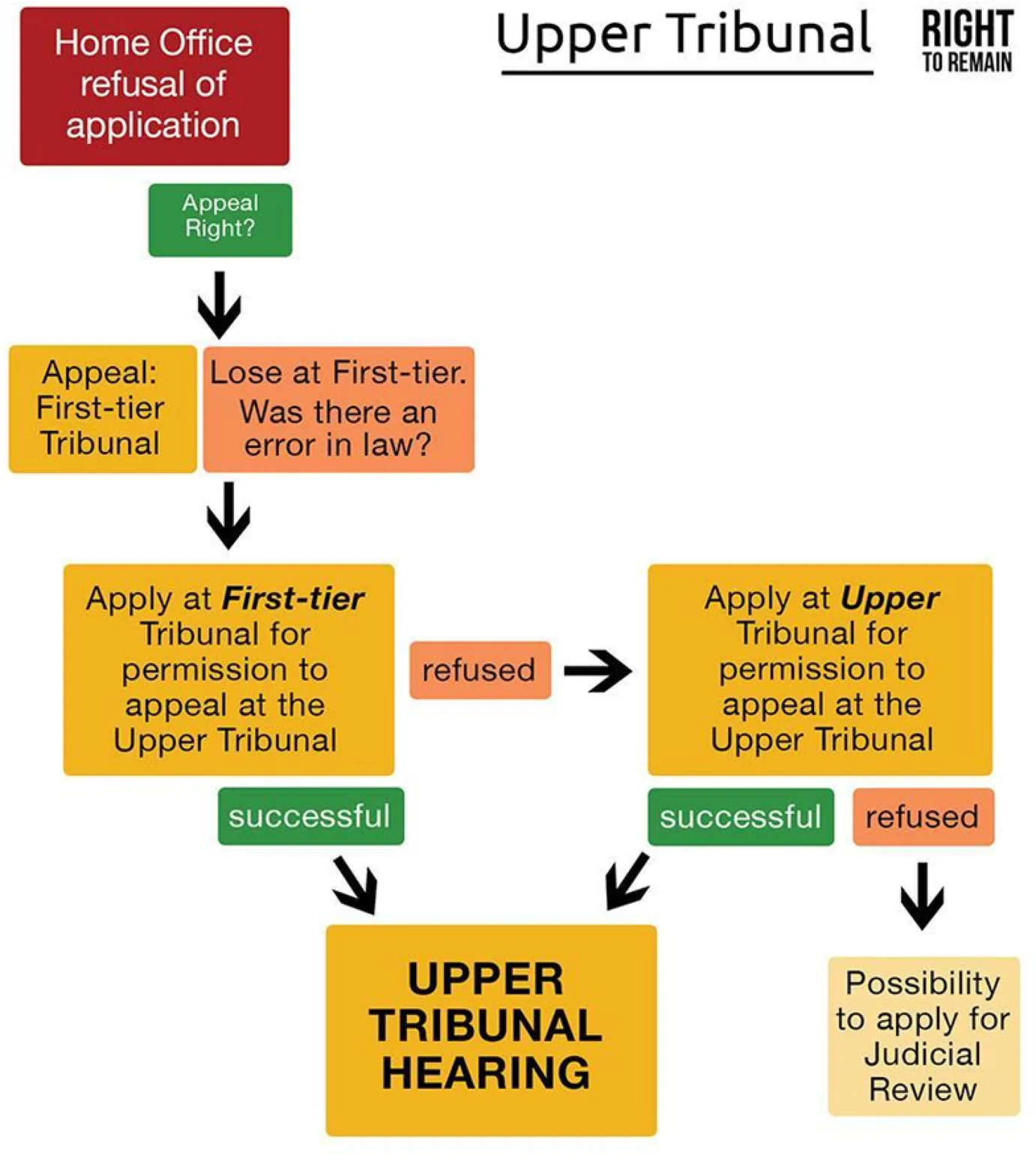
Flowchart illustrating the process of appealing at the upper-tier tribunal [72]

When an asylum applicant receives a negative decision from the Home Office following their substantive interview, their application proceeds as outlined in Fig. 3.

A DN is produced at the end of a first-tier and upper-tier tribunal and sent to the appellant. It must also be noted that DNs are published exclusively in English, calling into question whether appellants are able to even access the documents. For those who have legal representation, they presumably assist appellants in understanding the DN. For those 15% who are unrepresented, they may have to seek translation of the document independently, which would prove a difficult and potentially expensive task. It is therefore possible that applicants are never made aware of interpreting issues that occurred, leaving them unable to discuss or contest them.

The shift from substantive interview to appeal is a significant one in terms of physical setting and institutional requirements. Substantive interviews are conducted by civil servants in Home Office centres, but appeals take place in tribunals, where the setup resembles a criminal courtroom: the appellant sits on one side with their legal counsel, directly opposite the representative from the Home Office, and thus highlighting the adversarial nature of the setting [25]. The judge sits at the front of the room on a raised platform, which could be argued to represent their position ‘above the fray of the dispute’ [25]. Appeals are mostly undertaken in person, despite a move to video-conference during the pandemic, and are not audio-recorded [25].

Despite the formal legal setting, not all participants will necessarily be legal professionals [32]. Whilst a lawyer might represent the Home Office, it is more likely that they will be represented by a Home Office Presenting Officer (HOPO), which is a non-legal profession. The appellant’s representative might be accredited by one of four regulatory agencies, or the appellant may be unrepresented, which presents its own set of difficulties—roughly 15% of appeals are unrepresented [7]. The role of the judge now requires legal training, and is the role which receives the most authority in the room [25]. Judges’ treatment of interpreters varies between locations, with some being more patient and understanding than others. When judges are less patient, interpreters have been found to respond by altering speech to only translate questions directly posed to the appellant, omitting other conversations [25]. This set of varying institutional requirements and processes creates a complex adjudicatory framework [73], which is further complicated by the addition of interpreters [7].

Asylum appeals have long been recognised as problematic by practitioners and academics alike, often criticised as highly inconsistent decision-making processes which depend primarily on judges’ perceptions of appellants’ credibility, rather than a concrete legal framework [28, 32, 74]. The law is only one small piece of the puzzle in an asylum appeal; the broader social and institutional contexts, the individual personalities in the courtroom, and the interactions between them all play significant roles in the credibility assessment, and thus the decision-making process [7, 16, 17, 32, 61, 75–78]. Appeals are adversarial in nature; the judge focuses on credibility assessment and largely stays out of the questioning and fact-finding endeavours, which fall to the HOPOs and the appellant’s legal counsel [7, 25]. HOPOs will refer back to written records as evidence of oral testimony to search for discrepancies, and contestation to refugeehood will centre primarily on such consistency and credibility disputes [7].

Effective communication is understandably paramount to an appeal functioning successfully, and to ensure this, judges will often start proceedings by asking the interpreter to check the appellant understands them [25]. This strategy, however, is insufficient and does not do enough to ensure effective communication and interpreting (See Williamson [34] for alternative suggestions). Interpreting must take place in this setting which would already pose barriers to monolingual communication; interruptions, cultural and emotional barriers, and the management of several competing legal and non-legal identities in the tribunal [25]. As such, “more needs to be done to understand factors that influence decision-making in asylum contexts.” [7].

In summary, it is clear that interpreters must navigate conflicting demands, role expectations, and institutional language ideologies. The next Section outlines the methods used to investigate the extent to which this affects legal outcomes.

## Method

### Qualitative Content Analysis

Qualitative Content Analysis (QCA) is a useful technique for describing phenomena in documentary and textual data in particular [79–83]. QCA is helpful for ‘sorting things out’ in a research setting without a large amount of pre-existing knowledge [82]. QCA entails building a coding frame to systematically classify text into categories and themes [81], and it lends itself well to being combined with other methods [82, 83]. A mixed-method approach was thus adopted. An initial quantitative dimension offers a cross-Sectional perspective of asylum appeals and informs the coding process for the QCA, which reveals finer details. It is advantageous for the researcher to have direct experience of the research context themselves and increased familiarity with data [83]. It is therefore ideal for the present project, as I have worked in the immigration law field and am familiar with the institutional setting and processes.

### Quantitative Insight

**Fig. 4:**
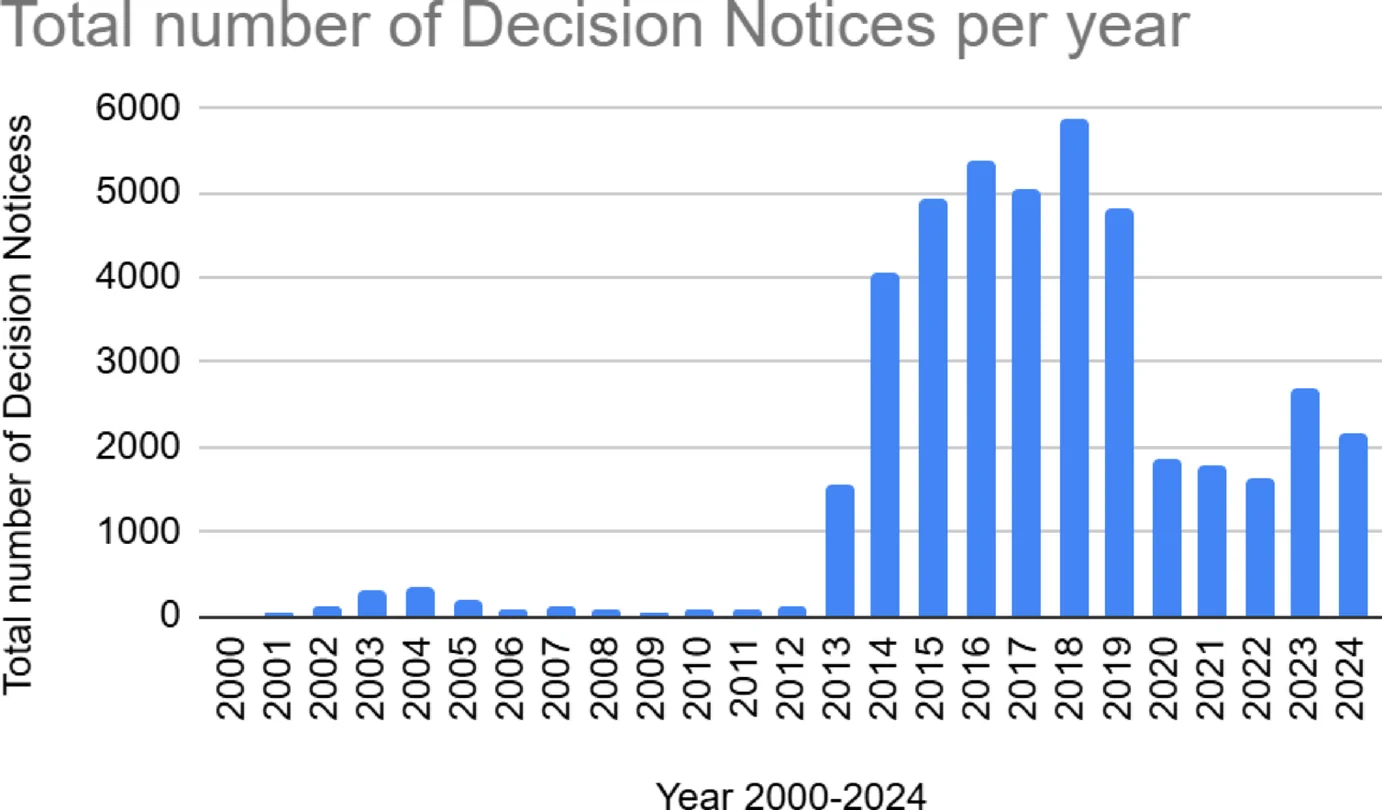
Bar chart of total number of Decision Notices per year

There are over 43,000 Decision Notices available to view online, dating from years 2000 to 2025 [85]. The number of DNs per year was first counted manually, revealing the trends in Fig. 4. There was a low number of DNs being produced (or at least published) for the first 12 years, followed by a slight increase in 2013. There was then a sharp increase between 2014 and 2019, and a dramatic decrease from 2020 to 2024. The latter period is perhaps explained by the Covid-19 pandemic, although further research would be required to confirm this.

**Fig. 5:**
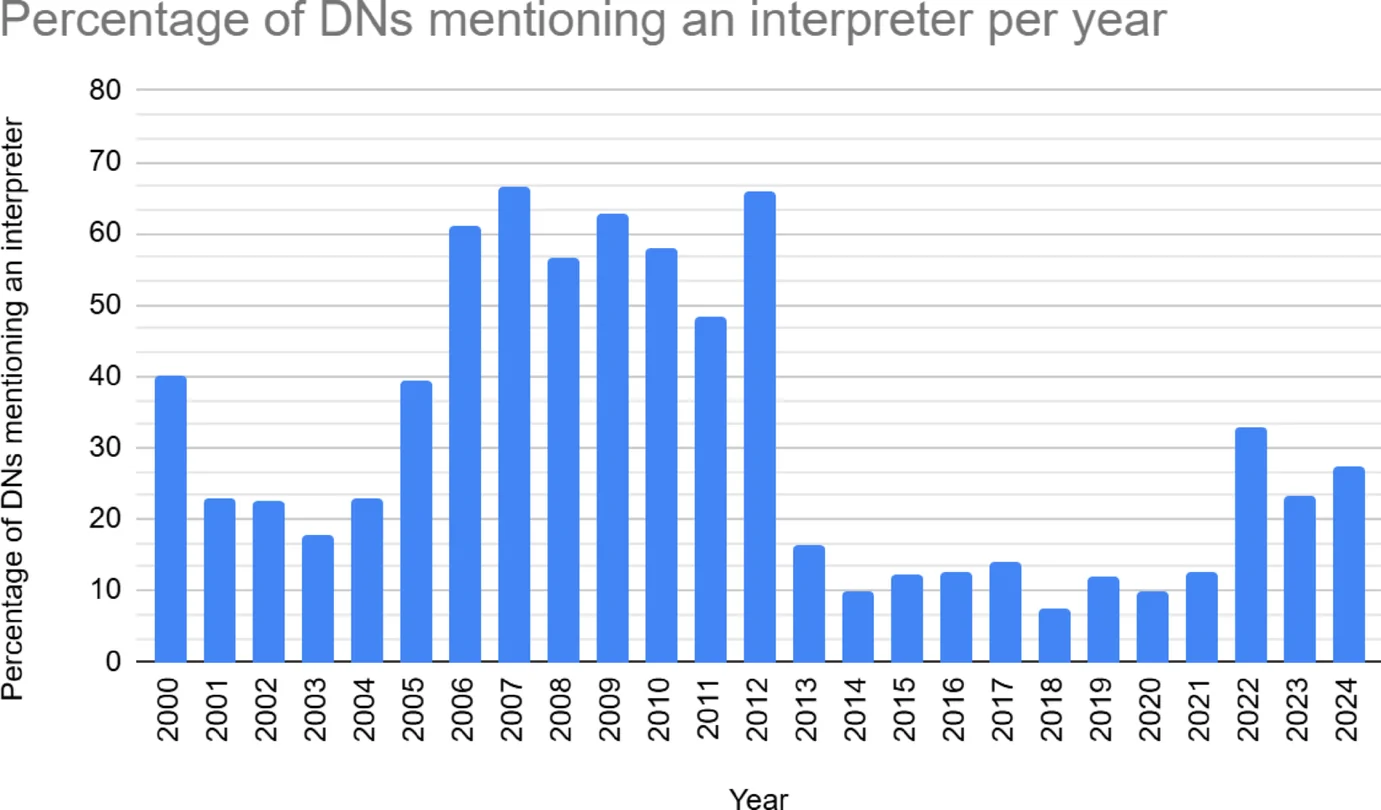
Bar chart of percentage of DNs mentioning an interpreter per year

The full dataset was then searched for DNs mentioning interpreters, using the Boolean operator ‘interpret’ to capture instances of the terms ‘interpreter’, ‘interpreting’ and ‘interpreted’. This search yielded over 6000 results. The same frequency-counting process was repeated for DNs mentioning an interpreter, which was compared to the results shown in Fig. 4, to reveal the proportion of DNs each year referring to an interpreter. Results are summarised in Fig. 5.

This analysis revealed an interesting pattern: the percentage of DNs mentioning an interpreter experienced a substantial increase between the years 2005–2012, followed by a sharp decline until the year 2022, when they slowly began to increase again. It is interesting to note the sharp decline in 2013, and whilst beyond the scope of this paper, future research might consider coinciding political events or operational changes.

The quantitative analysis helped determine a suitable time period for sampling for the qualitative analysis. It was clear that the number of DNs mentioning an interpreter was experiencing an increase in recent years. In line with the research aims to investigate the current impact of interpreting on asylum appeals, the sampling time frame was set from 2021 to 2024. The year 2021 was included so as to not exclude potentially valuable data; appeals processes are lengthy and it is possible that any factors affecting interpreting were set in motion before the increase seen in 2022.

### Qualitative Analysis

The qualitative analysis was performed by closely following the technique set out by Schreier [84].

#### Sampling

To collect the qualitative sample, simple random sampling (SRS) was used. SRS is a robust approach which improves representativeness of results [86] and complements QCA [82]. An online programme was used to generate random numbers corresponding to page numbers on the DN website.^1^This was a necessary step to reduce the large volume of data, as it would not have been possible to analyse 6000 reports. Each time a random number was generated, every DN from that page was examined to check its relevance. This was an important step because the search yielded some extraneous DNs, for instance, those including the term ‘interpret’ in another form (often referring to how a *law* was interpreted). Furthermore, the website also reports on non-asylum immigration appeals which are not relevant to the research aims, as interpreting functions quite differently in non-asylum immigration claims.

A sample size of 30 was deemed large enough to be representative of the large dataset, whilst remaining feasible within the constraints of the research [87, 88].

#### Building the Coding Frame

A coding frame was then built, in both a concept-driven and data-driven way [89]. A coding frame consists of main categories (also called dimensions) and sub-categories. The main categories were created deductively, based on the research aims. Three main categories were established:

1. *Type of interpreter reference* (in what way do judges mention/describe interpreters in the DN?)
2. *Impact level of the interpreting issue on the final appeal decision* (how does the issue they’ve outlined affect the material outcome of the case?)
3. *Final appeal decision* (was the appeal allowed or dismissed?)

QCA is systematic but flexible; once the coding frame has been built, it can be adapted as data is coded, and if new categories appear during the course of the analysis [89]. Although subcategories were initially proposed, it became clear that it would be necessary to approach these in a data-driven manner instead, as it was difficult to establish a priori the ways in which interpreters would be mentioned. Thus, the next stage was to read and reread each DN from the sample to see what subcategories were present. On the basis of this analysis, 12 subcategories were established, as shown in Table 1.

**Table 1:** Coding frame categories

Main categories	Type of interpreter reference	Impact level of the interpreting issue on the final decision	Final decision
Subcategories	(1) Presence acknowledged (2) Interpreter duties outlined (3) Problem: active (4) Problem: historic (5) Translation issue	(1) Procedural (2) Negligible (3) Substantial (4) Critical	(1) Allowed (2) Dismissed (3) Set aside and remade

#### Defining the Codes

The main categories ‘type of reference’ and ‘final decision’ and their corresponding subcategories were relatively straightforward to classify. The category of ‘impact level’, however, was more interpretively complex. After careful review of each of the 30 DNs, four operational definitions were established for each impact level (Table 2).

**Table 2:** Definitions of impact levels

Impact level	Definition
Negligible	The interpreter had little or no effect on the material outcome of the appeal. They are only briefly and inconsequentially mentioned in passing
Procedural	The interpreter is only mentioned in reference to the institutional process, for example ‘an interpreter was present’
Substantial	Whilst the interpreting issue was not itself grounds for the appeal, it did significantly affect the material outcome of the case
Critical	The interpreter or interpreting issue in question had such a profound impact on the appeal that it was the central basis of the appeal

To classify each impact according to the definitions, attention was paid to three key factors: (1) whether the interpreting issue was explicitly discussed as being *central* to the case, (2) whether the interpreting issue was linked to any *credibility* assessments of the applicant and (3) whether the interpreter issue was tied to any *material consequence* in the case. For instance, if an interpreter issue was discussed as affecting the procedural timeline, and/or in relation to the appellant’s credibility at any point in the case, this would be coded as ‘substantial’.

#### Analysis

Coding material to each category in the frame was an iterative process, with repeated visits and flexibility. For example, initially there was only one main category per DN, but it soon became clear that this was not suitable, as some DNs included multiple references to an interpreter. Thus the coding matrix was expanded to 34 total cases from 30 DNs, as four DNs included two distinct references to an interpreter. This avoided violating the principle of unidimensionality; in QCA, each main category should be mutually exclusive, and only represent a single characteristic with no conceptual overlap [89].

The coding frame was also amended following the appearance of several translation issues, which had initially been coded as ‘other’. Their prevalence led to the creation of a new subcategory ‘translation issue’. Similarly, there were initially only two subcategories for the ‘final decision’ dimension—‘allowed’ or ‘dismissed’—but it became clear from the data that a third was necessary, ‘set aside and remade’. This subcategory occurred frequently, and referred to decisions that had neither been allowed nor dismissed but had instead been returned to the previous stage of appeal for a fresh decision. My insight into the field assisted with the process of picking out relevant information through knowledge of legal terminology.

Once the trial coding phase was complete and the coding frame was finalised, the next step was to re-code the data for reliability. In a research team, members can code the data in parallel to check for consistency. When a researcher is working alone, as was the case for the present project, the researcher must step away from the data (in this case for 2–3 weeks) then return to re-code it de novo, to check for coding consistency [89]. Following the second coding round, there were eight discrepancies within main categories, out of the 34 total cases. The discrepancies were due to two factors: subtle differences between impact levels, and an increased familiarity with the data in the second round. For instance, the majority of discrepancies occurred between either substantial and procedural impacts, or substantial and critical impacts. Discrepancies were resolved by returning to the DN in question for a close inspection of the discrepancy. To establish the final codes, some were ‘graded up’ and some were ‘graded down’ in terms of impact level. For instance, extract 1 illustrates one such case.

“Ms [X] argued that when one looked at the transcript it could be seen that on a number of occasions **there were difficulties with the interpreter** and that perhaps gave a context to the judge’s interventions. It was a question of the appearance of unfairness rather than any actual bias. [...] [I]t is clear, as Ms [X] argued, that **there were issues of interpretation** which may have led the judge into taking a more assertive role in the hearing than she might otherwise have done. The questions she asked appear to me to be essentially matters, as she herself said in her comments, desiring clarification rather than seeking to find evidence to support a theory.”

Extract 1.^2^

In coding round 1, this reference to interpreting issues was coded as impact level ‘critical’ as interpreting difficulties were cited frequently throughout the report. In round 2, however, it was coded as ‘substantial’. Given the judge’s perspective that the interpreting issues were ‘matters’ rather than evidential, this was finally set at ‘substantial’. In addition, a further DN featured heavy discussion of interpreting issues, particularly surrounding a previous translation of the word ‘tourist’. This translation was relevant to the material evidence in the case, and to the appellant’s credibility (which centred on a type of visa they were using), as illustrated in Extract 2.

“There were points through the cross where Appellant 1 was not happy with the interpreter’s interpretation of parts of the answers. There was a question raised by the judge as to an interpretation of tourist and visit and the interpreter confirmed it was the same Baluchi word for both”.

Extract 2.^3^

This impact was increased from ‘substantial’ to ‘critical’ after careful consideration of the effect this issue had on the final decision. The re-coding process highlights the strengths of the QCA approach; it led to an enriched understanding of the data by repeated close inspection [81].

#### Ethics

Given the data’s context, ethical considerations were a priority throughout the methodological process. There was no formal ethical approval process, as the DNs are all published online and fully accessible to the general public. However, it is extremely important to maintain the privacy of the individuals discussed in the DNs. Many of them were already subject to anonymity orders and thus had names removed, but for those which did include names, these were strictly kept out of any data gathered for the research. Furthermore, any potentially identifiable points of discussion have been removed from this paper. A final important ethical consideration is the positionality of the researcher, which is discussed in Sect. 6.

## Results

### Coding Frequencies

In total, there were five appeals allowed, 11 dismissed, and 18 set aside and remade. In terms of impact levels, there were two references with critical impacts, 12 with a substantial impact, 18 with a procedural impact, and 1 with a negligible impact. Table 3 outlines the total frequencies of each impact level and final decision.

**Table 3:** Total frequencies of impacts and final decisions (the distribution of final decisions across each impact level)

Impact level	Count	Allowed	Dismissed	Set aside and remade
Critical	2	0	0	2
Substantial	13	1	5	7
Procedural	18	4	5	9
Negligible	1	0	1	0
Total	34	5	11	18

Aside from the one negligible impact, the majority of cases for *all* types of impact levels were set aside and remade. It is interesting to note the pattern of substantial impacts—only one of these 13 appeals (8%) were allowed, with most (7/13, 54%) being set aside and remade, and the rest being dismissed. In addition, for the two critical cases, neither of these were allowed, but instead set aside and remade. The decision to have an appeal set aside can be considered successful, although only inasmuch as the appeal has not been dismissed. Whilst the appellant has not been granted refugee status immediately, judges have at least acknowledged that there were sufficient grounds for appeal and the decision should be considered afresh. However, appeals where interpreting issues were not the focus—the procedural impacts—received a rather higher rate of allowals at 22% compared to 8% of cases where interpreters had a substantial impact on the case, although the overall subsample sizes are relatively small.

It is also interesting to note the results of the other main coding category: type of reference. Table 4 outlines the frequencies of each type of reference and the final decision in the case.

**Table 4:** Types of references for each impact level (numbers of each type of reference found within each impact level category)

Presence Acknowledged	19	0	2	17	0
Problem: Historic	6	2	3	1	0
Problem: Active	3	0	3	0	0
Translation issue	3	0	2	0	1
Interpreter duties outlined/instructions given	3	0	3	0	0
Total	34	2	13	18	1

The majority of procedural impacts came from simply acknowledging the interpreter’s presence. The solitary negligible impact came from a translation issue. For substantial impacts, these were fairly evenly distributed across each of the types of reference. The two critical impacts stem from historic problems in the case.

### Key Findings

The most important finding is that in the random sample of just 30 DNs, there were two critical and 13 substantial impacts of interpreting issues on final decisions. The results therefore reveal a clear answer to the research questions: judges are indeed referring to interpreters in a number of ways. The most frequent referral comes at a procedural impact, where judges acknowledge the interpreter’s presence. Judges also frequently refer to historic or active interpreting problems, where discrepancies or inaccuracies have been highlighted. The results also clearly demonstrate that interpreters are indeed having an impact on appeal outcomes and altering the decision-making process, demonstrating how important this research topic is. For instance, for both of the critical impacts, the appeal decisions were set aside and remade (Table 3). For all types of references that were active problems, these had a substantial impact (Table 4).

### Extracts from the Data

This Section provides extracts from the data to illustrate examples of the results.

#### Negligible Impact

In the one appeal which included a negligible impact, a question is raised around why a certain document was translated in Bangladesh rather than the UK:

“There is no reason why documents should be translated in any particular country or why translation in one country should be regarded as inherently preference [sic] to translation in another country. It all depends on the document. **Here nothing turns on the details of the translation**.”

Extract 3.^4^

This reference was coded as a ‘translation issue’ with a negligible impact, as the judge stated that ‘nothing turns on the details’ of the translation. The final decision in this case was a dismissal.

#### Procedural Impacts

The vast majority of procedural impacts came from the type of reference ‘presence acknowledged’ which was simply the judge outlining the fact that an interpreter had attended the hearing. For instance:

“The appellant attended the hearing of her appeal and gave evidence with the assistance of an **interpreter**.”

Extract 4.^5^

This reference was coded as: type of reference ‘presence acknowledged’, and impact level ‘procedural’. This final decision in this appeal was a dismissal.

#### Substantial Impacts

Extract 5 highlights an example of an interpreting issue that had a substantial impact:

“The appellant denied ever having instructed his solicitors to say that he has a blog. He did not know what blogging was, and even after several attempts by the **interpreter**, by me and by Mr [name], and then by his solicitor in re-examination, he clearly did not understand the concept. He is not a blogger.”

Extract 5.^6^

This reference was coded as ‘interpreter duties outlined/instructions given’ and impact level ‘substantial’. The interpreter here has been asked to step outside of the role of a conduit, and step in to explain a concept to the appellant. This understanding of the concept of blogging was used in part to assess the appellant’s credibility. It is therefore of substantial impact that the interpreter was tasked with an active role in the process of determining whether the appellant knew what ‘blogging’ is or not. This appeal was set aside and remade.

A further example of a substantial impact is shown in Extract 6.

“The appellant’s appeal was heard by First-tier Tribunal Judge McIntosh on 31 January 2020, following an initial start on 3 January 2020 which was discontinued due to difficulties understanding the **interpreter**.”

Extract 6.^7^

There was deliberation as to whether this ‘problem: historic’ reference should be coded as having an impact of ‘procedural’ or ‘substantial’. The final decision was to classify it as substantial, because it could be convincingly argued that an adjournment, and a subsequent delay in a hearing, allows either side more time to prepare their arguments for the appeal, and thus constitutes a potentially significant change to proceedings. This appeal was set aside and remade.

A further example of a substantial impact is evidence of the monoglot ideology [64], in Extracts 7a-b.

“The appellant had to give evidence through an interpreter, suggesting that she is more comfortable speaking Amharic than English, although I accept that she speaks some English.”

Extract 7a.^8^

Here the judge uses the presence of the interpreter as evidentiary material in determining the appellant’s identity. In this case, the judge argues that their limited ability to speak English suggests that they are more suited to a life in Ethiopia, along with their children:

“It is more likely than not that the children speak Amharic at home with their parents. But for any considerations to the contrary, the best interests of the children would be to return to Ethiopia, with their parents.”

Extract 7b.

The presence of the interpreter therefore had a substantial impact on the decision-making process in determining where the family should reside. Another DN contained similar information, seen in Extract 8.

“Despite claiming in her screening interview that Tigrinyan was her first language, before the FtTJ she resiled from that account stating “she did not say that Tigrinya was her main language, that was just written down on the record because there was a Tigrinya interpreter present”

Extract 8.^9^

Here the presence of an interpreter of a specific language is used as part of the credibility assessment, by means of contesting the appellant’s nationality. Extract 8 also highlights the role of interpreters in the narrator authorship and entextualisation ideologies [17, 49]; the interpreter’s presence caused an alteration to the written record and the assumed asylum narrative.

#### Critical Impacts

There were two cases which were coded as critical impact levels. The first (Extracts 9a-c) centred on an interpreter accuracy issue that the appellant had cited from their substantive interview.

“The appellant’s solicitors obtained the audio recording and it was placed before Mr [X]. In his statement he sets out how he conducted the process of producing the transcript and that he listened to the Home Office questions, the translation by the interpreter and the answers from the client. He recorded each part in the transcript to ensure consistency as to what was said. He stated that “he interpreted every word from the audio which he stated, “formed a clear and true picture of the information provided by the interview.””

Extract 9a.^10^

The appellant’s solicitors hired an expert witness; an interpreter who has been instructed to listen to the recording of the substantive interview, and produce his own transcript of it to check for any alleged inaccuracies. The DN continues (Extract 9b):

“The translation by Mr [X] reads “my wife, when I bought the stuff in the suite, I bought condom, I put it in my pocket but I forgot the bill receipt inside the bag.” In the statement made by Mr [X] he makes reference to the problems and interpretation which he describes as “one crucial point about the interpretation of the Home Office interpreter”. He identifies as follows “as interpreters, we are strictly expected use [sic] **first person interpreting**, because it is very confusing for all parties if second person interpreting is used. For example, if I say “he said I will write to you” you cannot tell if he is going to write or he meant that I will write on behalf of him to you. Therefore, it is a must for interpreters to use first person only to avoid confusing people. This problem was evident in his case, as the interpreter in the interview said “he said he put the receipt in the bag” which is unclear whether it means (the male client says, the other male person put the receipt in the bag) or (the male client says that he himself at the receipt in the back).”

Extract 9b.

This case highlights the importance of relaying by replaying rather than by displaying [58]. The original Home Office interpreter in the substantive interview failed to follow the interpreter’s code of conduct and used reported rather than direct speech. This error created confusion around who had put a receipt in a bag, an action which had been considered central to the appellant’s credibility by the judge in the first-tier appeal, who had dismissed the possibility of an interpreting error being the cause for this inconsistency. This was a clear case of a critical impact, as illustrated by Extract 9c:

“Looking at the translation, I accept the submission made by Mr [Y] that the record as set out in the interview [...] is inaccurate and that the account given by the appellant in the interview[...] is set out in those terms had it been properly interpreted. Furthermore, **it supports the appellant’s account that there was an interpretation error which was an explanation which was discounted by the judge who went on to find that the appellant had not given a consistent account concerning the factual basis of his claim**.”[...] “Even if it could be said that [...] the version given there was a different version from that given [...] this issue demonstrates that the appellant’s explanation for **the discrepancy as being as a result of “interpreter error” is correct**.”

Extract 9c.

In sum, the interpreter in this case had a critical impact on the outcome of this asylum application at the substantive interview stage. This led to the lodging of an appeal whereby legal counsel hired an expert witness to verify interpreting errors that had damaged the applicant’s credibility. This appeal was set aside and remade; it was returned to the first-tier tribunal for a fresh decision. As first-tier tribunal decisions are not available to the public, it is not possible to ascertain the current state of this appeal.

The second example of a critical impact hinges on a different type of interpreting error:

“The appellant said during the interview, and his representatives asserted several times afterwards to the respondent, that **the interpretation was unsatisfactory** and that the appellant had not had a proper opportunity to put his case forward. The respondent made no reply to those submissions other than to issue a decision rejecting the appellant’s asylum claim”

Extract 10a.^11^

Here the ‘respondent’ refers to the Home Office and the ‘interview’ refers to the substantive interview. Thus, the appellant claims that they had complained during their substantive interview that the interpretation provided was unsatisfactory, and that they reported this to the Home Office, who declined to respond to these claims, and instead issued a refusal notice for their asylum claim. The appellant then drafted an appeal against this refusal, to which the Home Office (the respondent) continued to fail to respond.

“The respondent had been directed by the First-tier Tribunal no fewer than nine times to respond specifically to the allegation about the interpreter at the interview.”

Extract 10b.

The appellant submitted their appeal argument, and the appeal hearing was scheduled to go ahead without representation from the Home Office, as they had continually failed to respond to the appeal argument.

“At the date fixed for the hearing, there was no attendance by or on behalf of the respondent. However, the hearing could not proceed, because **the interpreter booked by the First-tier Tribunal was not a Libyan Arabic interpreter**. The matter was adjourned, and the respondent was again directed to provide a review.”

Extract 10c.

The scheduled appeal did not go ahead, because the tribunal had booked the incorrect interpreter. This constitutes a second significant failing against the appellant, this time stemming from a lack of interpreter availability. When a second date was booked for the hearing, this time the Home Office attended. Following this second hearing, the appeal decision was ‘set aside and remade’. The interpreting issue was not the only critical piece of evidence, but its impact remains critical, as it did at least in part constitute the grounds for the appeal in this case.

## Summary

In sum, the results clearly suggest that interpreters are impacting appeal outcomes in several ways:

1. For cases where an interpreting issue had a critical impact on the outcome of the case, these always stemmed from historic problems in the case (see extracts 9a-c and 10a-c), and always received a decision of ‘set aside and remade’. However, caution must be taken in interpreting these conclusions because this category is only represented by a sample size of two, and other factors were also relevant to the outcome.
2. For cases where an interpreter had a substantial impact on the outcome of the case, this was reasonably likely to have come from any of the five types of interpreting issue, and the final decisions were most likely to be ‘set aside and remade’.
3. In the majority of cases where a judge mentioned an interpreter, it was to acknowledge their presence. Half of these cases with a procedural impact of an interpreting issue were set aside and remade, with the remaining half split between allowals and dismissals.

## Discussion

The quantitative analysis revealed that judges mention interpreters to a varying extent over time, with an increase in mentions since 2022. The results of the QCA show us what specific ways judges are talking about interpreters: for the most part, they are acknowledging their presence with a simple sentence. There were, however, several instances where judges described serious interpreting issues which affected the decision-making process and the outcome of the case.

In considering the impact of the findings, it is important to note the perspective that the DNs are written from. Judges write these reports and it is at their discretion what they include and what they omit. It is quite possible that interpreters play an even more crucial role in final decisions than appear in the results, or even less of one. This paper does not argue that this sample, or even the entire population of DNs, are representative of a ‘true’ picture of the asylum appeal system in the UK. The researcher’s own interpretation will always be present in the analysis, and the methodological process has been detailed to remain transparent. Data cannot ‘speak for itself’ [90], nor reveal unequivocal facts about the research setting [91]. Instead, the results highlight the existence of some empirical evidence of what the literature and practitioners in the field have suggested and intuited, and open the door for further research into this topic.

That being said, it is possible to make observations based on the results. The findings suggest that judges may primarily view interpreters as a procedural piece of the puzzle, although the data cannot determine whether this is an accurate portrayal or not. More importantly, though, the findings suggest that when judges acknowledge that an interpreting issue has critically impacted an asylum case, they may not be as willing to grant an allowal on that basis as they may be for other reasons. These findings therefore suggest that judges and decision makers need to be more aware of the crucial role that interpreters play, and the pivotal impact they can have on a case.

The findings show that interpreters are having significant impacts on appeal outcomes, and interpreting problems mostly comprise issues with interpreting at earlier points in the asylum cases. It is vital that future research uncover the mechanisms behind these issues: does the problem lie with interpreter training and qualification? Or stakeholder perceptions? Which specific linguistic features are causing the most problems? Are problems universal across languages and dialects?

The results presented here support the pervasiveness of the language ideologies [17, 49, 64] which have been seen to underpin the asylum system; they revealed how fundamental the inconsistency ideology is, and how paramount consistency and credibility are in the decision-making process. The results also highlighted clear instances of the monoglot, narrator authorship and entextualisation ideologies at play (see Extracts 7 and 8). Future research should consider more closely the relationship between language ideologies, interpreters, and asylum outcomes; for instance, are interpreters encouraged to adopt these ideologies by stakeholders?

In addition, the publicly accessible database of DNs contains a wealth of information that could be exploited further by future research. Each DN lists other factors which could impact the results, for instance, whether the hearing was in-person or remote, who the presiding judge was, where the hearing centre was located, what the second language was, and many others which might affect the relationship between interpreters and appeals. Given the individuality and flexibility associated with appeals, different tribunal centres often lead to different sets of results [28].

Furthermore, the data only comes from upper-tier tribunals, whereas many asylum appeals will only reach the first-tier, where DNs are inaccessible to the public. It could be that interpreting is either more or less problematic at the first-tier, or that different types of interpreter issues arise there. In addition, there are several important voices missing from this study. It would be valuable to triangulate the findings with interviews from those involved in the appeals under study; appellants, legal counsel, and decision makers. This could reveal important insights about the way interpreting issues and impacts are perceived by those directly affected by them.

Future research might also expand upon the initial quantitative investigation of trends over time with DNs, and consider, for instance, mapping the fluctuations against events during the corresponding time period, to see what these patterns might reveal about interpreters and appeals.

Whilst the sample size in this study is relatively small, there are tens of thousands of appeals heard and decided each year in the UK (see Sect. 1) and it is crucial that we investigate how many of their decisions are impacted by interpreting issues.

## Limitations

It is important to acknowledge limitations to the study [92]. Conducting a QCA inevitably entails making choices about what is and isn’t included in the coding frame. This is important because it allows the researcher to reduce a large amount of material, but it also removes potentially important pieces of information, such as the contextual factors discussed above [80].

Furthermore, qualitative researchers bring their own positionality, background and values to their research, and it is important to acknowledge that the analysis will always be influenced by these to some degree [93]. My own position has been shaped by my experience working as a caseworker at an immigration law firm, where I witnessed many clients face interpreter issues. This has given me an insider perspective on institutional processes, which could predispose me to biased interpretations of the data, though I have worked to ground my analysis in the textual evidence rather than my own assumptions. My research is driven by concerns around access to justice for marginalised people navigating legal systems, which, along with my academic background in forensic linguistics and social justice have also informed my positionality. It is important to acknowledge my position as an outsider to the lived experiences of asylum applicants. I do not presume to speak on behalf of this community, but am committed to contributing empirical evidence about how interpretation issues function within asylum appeal adjudication. This is not necessarily a limitation per se, but an important point to acknowledge nonetheless.

## Conclusion

This analysis, based on an innovative combination of quantitative and qualitative methods, has revealed clearly that interpreters are having a significant impact on appeal outcomes. Whilst the study cannot tell us the exact steps that can be taken to improve practice, it can draw attention to empirical data showing that it *does* happen. It is the hope of this paper that decision makers acknowledge these findings and use them to inform and improve practice. This study does not aim to criticise interpreters. The issues identified reflect institutional problems, not individual failings. Since the outsourcing of services in 2011, many qualified interpreters have simply left the profession. Low pay and poor conditions have driven out expertise. The current system places enormous pressure on those who remain. These are structural problems requiring structural solutions. There is clearly a serious need for a reform to interpreting services. We should bear in mind, however, that even if interpreting is improved, there will still be inconsistencies, because that is the very nature of translation. The findings are a step in the right direction for improving access to justice, but more important is (a) that more research is done to pinpoint the mechanisms behind the findings and (b) that asylum decision makers acknowledge that interpreters affect outcomes, and factor this into their decision-making process. Interpreters can make or break asylum appeals—a simple linguistic choice might determine whether someone gets refugee protection or faces deportation.

## Data Availability

The datasets analysed are available in the UK Upper Tribunal (Immigration and Asylum Chamber) repository, https://tribunalsdecisions.service.gov.uk/utiac

## Footnotes

1 https://tribunalsdecisions.service.gov.uk/utiac.

2 DN Reference: DN UI-2022-001797. This reference can be used to search the database, available at: https://tribunalsdecisions.service.gov.uk/utiac.

3 DN reference: UI-2023-000717, UI-2023-000718, UI-2023-000719, UI-2023-000720.

4 DN reference: PA/11608/2019.

5 DN reference: PA/52957/2021.

6 DN reference: PA/10203/2019.

7 DN reference: PA/07732/2019 (V).

8 DN reference: PA/04171/2019.

9 DN reference: PA/01330/2020 (V).

10 DN reference: PA/06881/2019 (V).

11 DN reference: UI-2022-005510.
